# Multicenter Cross-sectional Study of Patients with Rheumatoid Arthritis in Greece: Results from a cohort of 2.491 patients

**DOI:** 10.31138/mjr.29.1.27

**Published:** 2018-03-19

**Authors:** Konstantinos Thomas, Argiro Lazarini, Evripidis Kaltsonoudis, Alexandros Drosos, Ioannis Papalopoulos, Prodromos Sidiropoulos, Pelagia Katsimbri, Dimitrios Boumpas, Panagiota Tsatsani, Sousana Gazi, Kalliopi Fragkiadaki, Maria Tektonidou, Petros P. Sfikakis, Lina Pantazi, Kyriaki A. Boki, Eleftheria P. Grika, Panagiotis G. Vlachoyiannopoulos, Konstantina Karagianni, Lazaros I. Sakkas, Theodoros Dimitroulas, Alexandros Garyfallos, Dimitrios Kassimos, Gerasimos Evangelatos, Alexios Iliopoulos, Maria Areti, Constantinos Georganas, Konstantinos Melissaropoulos, Panagiotis Georgiou, Periklis Vounotrypidis, Konstantinos Ntelis, Clio P. Mavragani, Ilias Bournazos, Gikas Katsifis, Christos Mavrommatis, George D. Kitas, Dimitrios Vassilopoulos

**Affiliations:** 1 Joint Rheumatology Program, National and Kapodistrian University of Athens, School of Medicine, Athens, Greece,; 2 Rheumatology Clinic, University of Ioannina, Ioannina, Greece,; 3 Clinical Immunology and Allergy Department, University of Crete, Heraklion, Greece,; 4 Rheumatology Unit, KAT Hospital, Athens, Greece,; 5 Rheumatology Unit, Sismanoglio Hospital, Athens, Greece,; 6 Department of Rheumatology, University of Thessaly, Larissa, Greece,; 7 4^th^ Department of Medicine, Aristotle University, Thessaloniki, Greece,; 8 401 General Military Hospital, Athens, Greece,; 9 Rheumatology Unit, NIMTS Hospital, Athens, Greece,; 10 Private Practice, Greece,; 11 Rheumatology Unit, Agios Andreas Hospital, Patras, Greece,; 12 Rheumatology Unit, Navy Hospital, Athens, Greece,; 13 Hygeia Hospital, Athens, Greece

**Keywords:** rheumatoid arthritis, comorbidities, biologics, methotrexate, vaccination, infections, tuberculosis, hepatitis, cardiovascular diseases

## Abstract

**Aim of the study:**

To evaluate the current disease characteristics, treatment and comorbidities of rheumatoid arthritis (RA) in Greece.

**Methods:**

Multicenter, cross-sectional study with a 9-month recruitment period between 2015 and 2016. Demographics, disease characteristics, treatment and comorbidities were collected via a web-based platform.

**Results:**

2.491 RA patients were recruited: 96% from tertiary referral centers, 79% were females with a mean age of 63.1 years and disease duration of 9.9 years. Fifty-two percent were rheumatoid factor and/or anti-CCP positive, while 41% had erosive disease. Regarding treatment, 82% were on conventional synthetic disease modifying anti-rheumatic drugs (csDMARDs), 42% on biologic DMARDs (TNFi: 22%, non-TNFi: 20%) and 40% on corticosteroids (mean daily dose: 5.2 mg). Despite therapy, 36% of patients had moderate and 12% high disease activity. The most frequent comorbidities were hypertension (42%), hyperlipidemia (33%), osteoporosis (29%), diabetes mellitus (15%) and depression (12%). Latent tuberculosis infection (positive tuberculin skin test or interferon gamma release assay) was diagnosed in 13 and 15.3% of patients, respectively. Regarding chronic viral infections, 6.2% had history of herpes zoster while 2% and 0.7% had chronic hepatitis B and C virus infection, respectively. A history of serious infection was documented in 9.6%. Only 36% and 52% of the participants had ever been vaccinated against pneumococcus and influenza virus, respectively.

**Conclusion:**

This is one of the largest epidemiologic studies providing valuable data regarding the current RA characteristics in Greece. Half of patients were seropositive but despite therapy, half displayed residual disease activity, while preventive vaccination was limited.

## INTRODUCTION

Rheumatoid arthritis (RA) is one of the most common autoimmune diseases in the general population that affects mainly middle- or advanced aged women with a worldwide prevalence of 0.5–1%.^1,2^ If not timely and appropriately treated, it can lead to progressive joint destruction, functional disability and increased morbidity and mortality.^1^ During the past 20 years, the introduction of biologic therapies in daily clinical practice for patients who have failed treatment with conventional synthetic DMARDs (csDMARDs), resulted in better control of disease activity, prevention of permanent damage and decreased mortality.^1^ There is limited information on the prevalence and disease characteristics of RA in Greece. The estimated prevalence in Greece ranges between 0.35 to 0.68%,^3–5^ a finding that is concordant with the estimates from other Mediterranean countries.^6^ Regarding current therapies, in the largest Greek study so far conducted by the Hellenic Registry of Biologics, 1.028 RA patients treated with tumor necrosis factor (TNF) inhibitors (TNFi) were included.^7^ The primary purpose of the study was to evaluate the drug survival and the serious complications of TNFi during long-term follow-up (2004–2009). A recent single-center study from Greece has focused mainly on the infectious complications of DMARD therapy (both cs- and biologic, b-DMARD) in RA patients^8^ while other recent studies have focused on drug survival and side effects of different bDMARDs.^9,10^ Although these studies provided useful data regarding bDMARD survival^7,9,10^ and DMARD complication rates,^7–10^ a number of unanswered questions regarding the current disease activity, severity, functional status, serologic profile, therapeutic patterns and prevalence of comorbidities of RA in Greece and worldwide remain.

We have designed a 3-year-long prospective, multi-center (including hospital and private practices) study of RA patients in Greece. Here, we present the results of the first phase which was a cross-sectional study of baseline patient and disease characteristics, treatment and comorbidities of RA in Greece.

## MATERIAL AND METHODS

### Patients

This was a multicenter, prospective study held by the RA Study Group of the Greek Rheumatology Society. Participating centers included academic and non-academic rheumatology clinics, National Health System out-patient clinics and private offices. Ethical approval was provided by the local institutional boards of participating centers and informed consent was provided by all patients before their inclusion in the study. The design of this prospective study includes three successive phases, as described below. Until today, the first two phases of the study have been completed and the main results of phase 1 are presented here.

During phase 1, a cross-sectional evaluation of RA patients seen during a 9 month recruitment period in each center, clinic or office was performed (the whole recruitment period lasted from June 2015 to September 2016). All patients recruited during this phase formed the working cohort of the study. Participating physicians were entering data either through a printed form or electronically via a specific web form through a designed portal (www.rheumstudygrps.gr).

This form contained the following information for each patient:

#### 1. Patient and disease characteristics:

Patients’ demographics included: age, gender, weight, height, working status (active worker, unemployed, retired), educational status (the highest level of education achieved), smoking and alcohol habits. Disease activity was assessed by the DAS28 (Disease Activity Score using 28 joints)–erythrocyte sedimentation rate (ESR) score while function was assessed by the Health Assessment Questionnaire (HAQ). The serological status (presence or absence of rheumatoid factor-RF and anti-cyclic citrullinated peptide antibodies-anti-CCP) of the patients was also recorded. The severity of the disease was evaluated by the presence or absence of erosions in plain joint X-rays (physician reported), history of joint arthroplasties and RA-related interstitial lung disease.

#### 2. Treatment patterns

For each patient past and current use of anti-rheumatic medications (including current dose and reasons for previous discontinuation) was also recorded. Anti-rheumatic medications included non-steroidal anti-inflammatory drugs (NSAIDs), analgesics, corticosteroids (CS) and DMARDs either csDMARDs or bDMARDs.

#### 3. Comorbidities

The following comorbidities were recorded: hyperlipidemia (documented by the use of hypolipidemic therapy), coronary artery disease (documented by the use of therapy and/or history of stable angina, acute coronary syndrome or angioplasty/coronary artery bypass surgery), cerebrovascular disease (documented by the use of therapy and/or history of thrombotic or hemorrhagic stroke), peripheral vascular disease (documented by the use of specific medical therapy or history of revascularization), diabetes mellitus (documented by the use of antidiabetic medications and/or insulin), chronic obstructive pulmonary disease (COPD, with or without use of oxygen therapy at home), arterial hypertension (documented by the use of anti-hypertensive medications), depression (documented by the use of anti-depressants), osteoporosis (documented by the use of anti-osteoporotic therapies and/or history of osteoporotic fractures), current or past hepatitis B virus (HBV) infection (documented by the specific serology including HBsAg, anti-HBc and anti-HBs antibodies), current or past hepatitis C virus (HCV) infection (documented by anti-HCV antibodies and HCV-RNA testing), history of tuberculosis (TB) or latent TB infection (LTBI), documented by a positive tuberculin skin test (TST) or an interferon-gamma release assay (IGRA), history of herpes zoster (HZ), current or past history of neoplastic diseases and history of vaccination against influenza (in the last year or in the past), (ever) and HBV (ever).

Phase 2 started one year after the initial evaluation and lasted also 9 months (whole period of recruitment: June 2016 – September 2017). During this phase, patients from the initial working cohort were re-evaluated 12 months after their first visit and a new set of data were collected using the same methods that were used in phase 1 (printed or web-based forms). During this phase, new patients or patients not previously entered in the working cohort were also recruited and formed the validation cohort of the study.

Phase 3 will be the last phase of the study (June 2018–September 2019) and it will include the third evaluation of the working cohort (3 years after the 1^st^ and 2 years after the 2^nd^ evaluation) as well as the 2nd evaluation of the validation cohort (2 years after its 1^st^ evaluation).

### Statistical analysis

All analyses were performed with the use of Microsoft Excel 2013 and IBM SPSS Statistics v.20 software. Data were analyzed by descriptive statistics. Demographic and descriptive continuous variables are expressed as mean (standard deviation, SD) and median values (interquartile range, IQR). Categorical variables are expressed as percentages. Chi square or Fisher’s exact test was used for comparison of dichotomous and Mann-Whitney or t-test for continuous variables. Binary logistic regression analysis was performed in order to identify variables associated with bDMARD use and history of serious infections.

## RESULTS

### Patient characteristics

During phase 1 of the study, 2.491 RA patients were recruited. The majority of patients were seen at hospitals (96%, 2401/2491); 79% were women with a mean age of 63 years (**Table 1**). Regarding their working status, only 28% of patients were actively working at the time of the evaluation while 19% were unemployed and more than half (53%) had retired from their work. Even among patients at working age (< 67 years old), one-third had already retired (35%) and only 41% were still employed. More than half of patients (54%) were high or university/higher technical school graduates (37% and 17%, respectively). Sixty-eight percent of patients had an increased BMI being either overweight (42%) or obese (26%). One-third of patients (35%) were either active or past smokers (19% and 16%, respectively) and also one-third (33%) reported current alcohol use (6% of them more than twice a week).

**Table 1.** Patient and disease characteristics.

**Variable**	

*Patient characteristics*	

n	2.491

Age, years	63.1 ± 13.1 (64.6)

Females, %	79%

BMI (Kg/m^2^), (n=2.045)	
Underweight (<18.5)	1%
Normal (18.5–24.9)	30%
Overweight (25–29.9)	42%
Obese (>30)	26%

Smoking habits, % (n=2.395)	
No history of smoking	65%
Active smokers	19%
Past smokers	16%

Alcohol use, % (n=2.408)	
No active drinking	67%
≤ 1 time/month	16%
2–4 times/month	11%
2–3 times/week	4%
≥ 4 times/week	2%

Educational status, % (n=2.035)	
Preliminary school graduate	46%
High school graduate	37%
University or higher technical graduate	17%

Working status, % (n=2.194)	
Employed	28%
Retired	53%
Unemployed	19%

*Disease characteristics*	

Disease duration, years (n=2.236)	9.9 ± 8.7 (7)

Early RA (< 2 years), n (%) (n=2.236)	237 (11%)

RF and/or anti-CCP+, n (%) (n=2.342)	1.214 (52%)

DAS28-ESR (n=2.126)	3.4 ± 1.3 (3.1)

HAQ (n=2.075)	0.48 ± 0.56 (0.25)

Joint erosions (X-rays, n=1.888)	779 (41%)

History of joint arthroplasties (n=2.491)	231 (9.3%)

RA-associated interstitial lung disease (n=2.364)	127 (5.4%)

Data are expressed as mean ± 1 Standard Deviation (median), unless otherwise specified.

In parenthesis the number of patients with available data from the whole cohort is also shown.

BMI, body mass index; RA, rheumatoid arthritis; DAS28-ESR, Disease Activity Score in 28 joints; ESR, erythrocyte sedimentation rate; RF, rheumatoid factor; Anti-CCP, anti-cyclic citrullinated peptide antibodies; HAQ, Health Assessment Questionnaire.

### Disease characteristics and severity

The mean disease duration at the time of evaluation was approximately 10 years (9.9 ± 8.7 years); only 11% of the patients had early disease (<2 years disease duration). Approximately half (52%) of patients in this cohort were RF and/or anti-CCP positive (**Table 1**).

**Figure 1.**
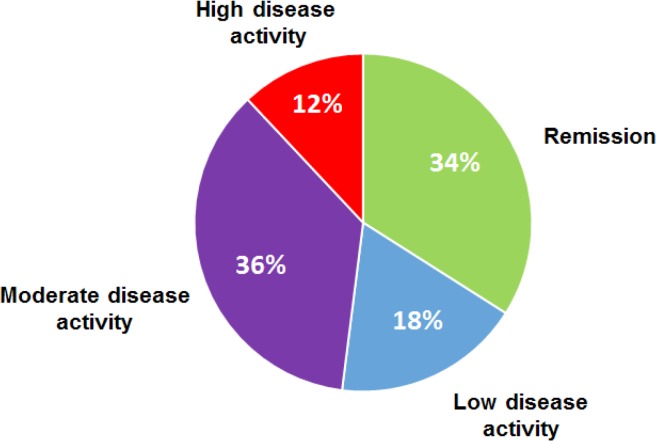
Current RA disease activity according to DAS28-ESR score. The current disease activity categories (%) of the RA cohort according to the Disease Activity Score 28 by the erythrocyte sedimentation rate (DAS28-ESR) is shown.

Data on disease activity were available for 2.126 (85%) patients. Overall, the mean DAS28-ESR score at the time of evaluation was 3.4 ± 1.3 indicative of moderate disease activity. For the 1.457 patients for which DAS28-ESR was available both at diagnosis and at current evaluation, a significant decrease in disease activity was noted (from: 4.9 ± 1.1 to 3.1 ± 1.1, p < 0.0001). However, despite this improvement, almost half of patients (48%) had still either moderate or high disease activity (36% and 12%, respectively, **Figure 1**).

Patients’ HAQ was available in 2.075 patients (83%) with a mean value of 0.48 ± 0.56 (**Table 1**). HAQ ≥ 1 was recorded in 22% of patients (n=451). Compared to the HAQ at diagnosis there was also significant improvement (from: 1.53 ± 0.98 to 0.40 ± 0.5, p < 0.0001, paired data available for 1.301 patients).

Regarding disease severity, erosive disease (physician reported by X-rays) was present in 41% of patients while 9.3% of patients had a previous history of joint arthroplasty (**Table 1**) and 5% had RA-associated interstitial lung disease (n=127).

### Current treatment

**Figure 2.**
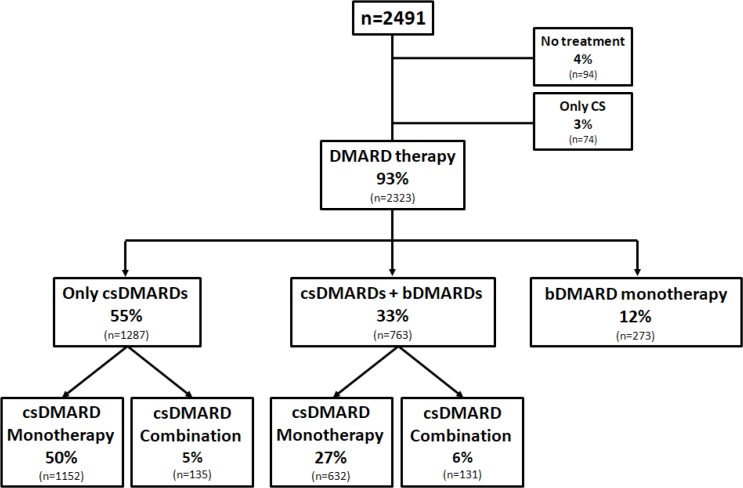
Treatment flow chart of the RA cohort. The therapeutic patterns of the 2491 RA patients is depicted. DMARD, disease modifying anti-rheumatic drug; csDMARDs, conventional synthetic DMARDs; bDMARD, biologic DMARDs

As shown in **Table 2** and **Figure 2**, the majority of patients at the time of evaluation were on treatment (n=2.397, 96%). The vast majority of patients were receiving DMARDs (n=2.323, 93%) either cs- (n=2.050, 88%) and/or b-DMARDs (n=1.036, 45%). Ninety-four patients were off therapy (4%) while 74 were using only CS (3%). Overall, 40% of patients (n=985) were on CS with a mean prednisolone daily dose of 5.2 ± 3.5 mg (**Table 2**). NSAIDs and simple analgesics were used by 7% and 21% of patients, respectively.

**Table 2.** Treatment characteristics.

**Therapy**	**n**	**%**
**No therapy**	94	**4%**
**Corticosteroids only**	74	**3%**
**DMARD therapy**	2323	**93%**
csDMARDs	2050	**82%**
*bDMARDs*	1036	**42%**
**Corticosteroids**	985	**40%**
Daily dose in mg, mean ± 1 S.D. (median)	**5.2** ± **3.5 (5)**	
**NSAIDs**	183	**7%**
**Analgesics**	536	**21%**

The treatment patterns of the patient cohort is shown. DMARD, disease modifying anti-rheumatic drug; csDMARDs, conventional synthetic DMARDs; bDMARDs, biologic DMARDs; S.D., standard deviation; NSAIDs, non-steroidal anti-inflammatory drugs

#### csDMARDs

**Figure 3.**
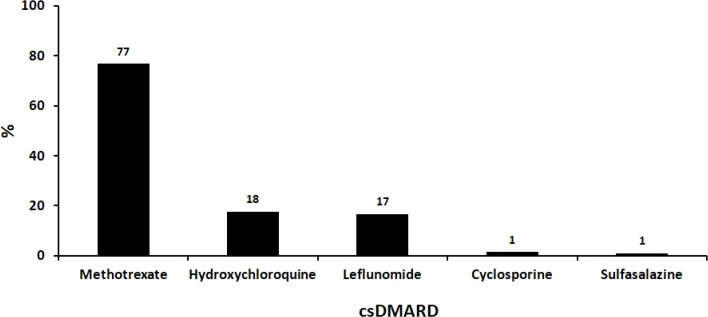
Conventional synthetic DMARD use in RA patients. The % use of the different conventional synthetic disease modifying anti-rheumatic drugs (csDMARDs) is shown (n=2.050). Some patients used combination of csDMARDs (n=266, see figures 2 and 4) and thus the cumulative sum exceeds 100%.

Among patients treated with DMARDs (n=2.323), 88% were on csDMARDs (n=2.050), given either as mono-therapy (n=1.287, 55%) or in combination with bDMARDs (n=763, 33%, **Figure 2**). As shown in **Figure 3**, the most commonly used csDMARD, was methotrexate (MTX, 77%) followed by hydroxychloroquine (HCQ, 18%) and leflunomide (LEF, 17%). The mean dose of MTX was 13.2 ± 3.6 mg/week (median: 12.5 mg/week).

**Figure 4.**
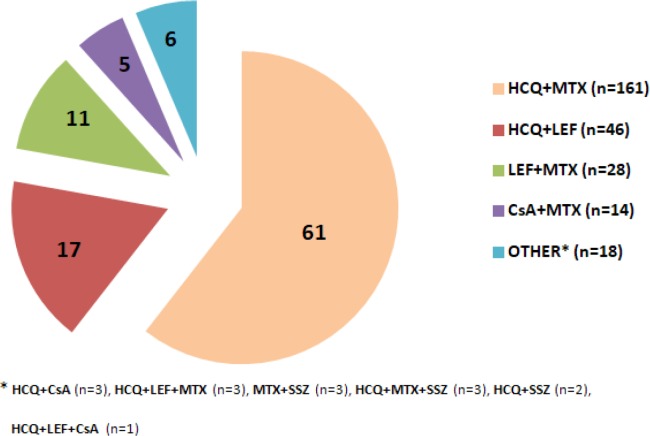
Combination csDMARD therapy patterns. The different combinations of conventional synthetic disease modifying anti-rheumatic drugs (csDMARDs, n=266, see also figure 2) used in the RA cohort is shown in %. HCQ, hydroxychloroquine; MTX, methotrexate, LEF, leflunomide; CsA, cyclosporine; SSZ, sulfasalazine.

csDMARD combination was used only in 11% of patients (n=266, **Figures 2** and **4**). Among patients using combination csDMARDs, the most common combination was that of HCQ plus MTX (61%) followed by HCQ plus LEF (17%) and MTX plus LEF (11%, **Figure 3**). The triple combination therapy of MTX, LEF and SSZ was used only by 3 patients (1.1%).

#### bDMARDs

bDMARDs were administered in 1.036 patients in this RA cohort either as monotherapy (n=273, 12%) or most commonly in combination with csDMARDs (n=763, 33%).

Compared to patients who were receiving only csDMARDs (n=1.287, **Figure 2**), bDMARD treated patients (n=1.036) were younger (mean age: 61.5 vs. 64.1 years, p<0.0001), more commonly females (83.6% vs. 77.1%, p<0.0001) and RF and/or anti-CCP + (58.7% vs. 47.6%, p<0.0001) with higher DAS28-ESR (5.2 ± 2.5 vs. 4.7 ± 1.1, p<0.0001) and HAQ (1.7 ± 1.1 vs. 1.4 ± 0.1, p<0.0001) at diagnosis while they tended to have longer disease duration (mean duration: 12.5 vs. 7.8 years, p<0.0001) and more severe disease as evidenced by the more common presence of erosions (56.3% vs. 30.1%, p<0.0001) and history of arthroplasties (11.4% vs. 7.5%, p=0.001). In terms of csDMARD use, bDMARD-treated patients used less often HCQ (8.4% vs. 21.4%, p< 0.0001) and MTX (55.5% vs. 77.5%, p<0.0001) compared to patients treated only with csDMARDs. No statistically significant differences in BMI, smoking, alcohol use, educational status, presence of interstitial lung disease or corticosteroid use was noted between the two groups.

In multivariate analysis, younger age (OR=0.972, 95% confidence intervals-C.I.=0.959–0.989), longer disease duration (OR=1.037, 95% C.I.=1.018–1.057), seropositivity (OR=1.462, 95% C.I.=1.094–1.953), erosive disease (OR=2.324, 95% C.I.=1.682–3.210) and a high HAQ at diagnosis (OR=1.243, 95% C.I.=1.025–1.507) were associated with bDMARD use.

**Figure 5.**
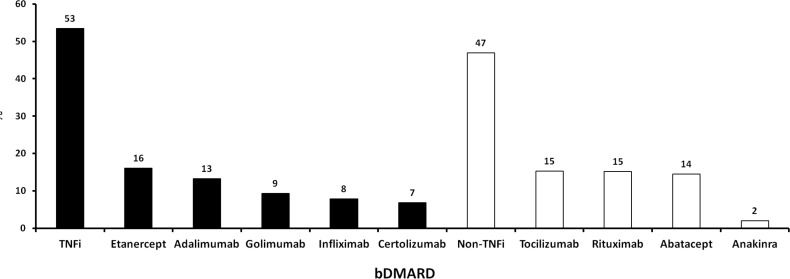
Biologic DMARD use in RA patients. The % use of the different biologic disease modifying anti-rheumatic drugs (bDMARDs) is shown (n=1.036, see also figure 2). TNFi, tumor necrosis factor inhibitors

In **Figure 5**, the specific type of bDMARD administered is shown. More than half of patients (n=554, 53%) were on TNFi and the remaining on non-TNFi treatment (n=486, 47%). For approximately half of bDMARD treated patients (n=506, 49%), this was their 1st biologic, in 25% (n=262) their 2nd, in 12% (n=127) their 3^rd^ while 14% (n=141) had used ≥3 bDMARDs previously.

#### Comorbidities

##### Cardiovascular diseases

**Figure 6.**
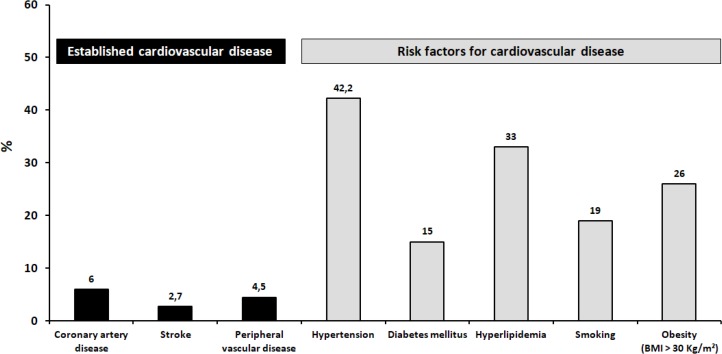
Prevalence of established cardiovascular disease and its risk factors. The prevalence (%) of the established cardiovascular disease and its different risk factors in the whole RA cohort (n=2491) is shown. BMI, body mass index.

A history of coronary artery disease, stroke and peripheral vascular disease (based on definite history and/or therapy) was reported in 6%, 2.7% and 4.5% of patients, respectively (**Figure 6**). The prevalence of cardiovascular disease risk factors was also high. Specifically, arterial hypertension was reported in 42%, hyperlipidemia in 33%, obesity (BMI > 30 kg/m^2^) in 26% and diabetes mellitus in 15% of patients whereas 19% of participants were active smokers at the time of registration. Overall, 40% of patients had at least two of the aforementioned cardiovascular disease risk factors.

##### Hospitalizations - Infections

A history of hospitalization during the last 12 months was documented in 7.1% of patients (199 hospitalizations in 182 patients). From the main prespecified causes, infections accounted for almost one third of admissions (60/199, 30%) and various cardiovascular events for 8.5% (17/199) of all hospitalizations.

**Figure 7.**
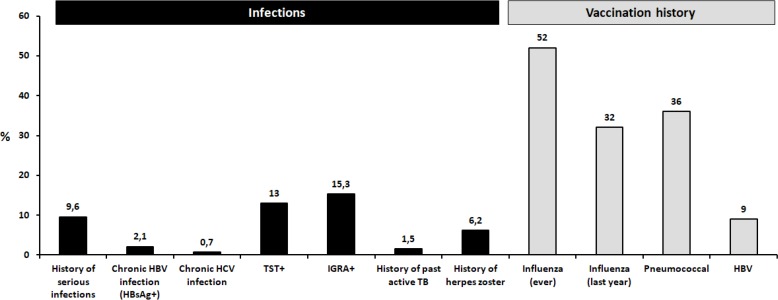
Prevalence of infections and vaccinations in the RA cohort. The prevalence (%) of different infections and vaccinations against common pathogens such as influenza and hepatitis B virus (HBV) and pneumococcus is depicted. HBsAg, hepatitis B surface antigen, HCV, hepatitis C virus; TST, tuberculin skin testing; IGRA, interferon gamma release assays; TB, tuberculosis.

A history of serious infection (defined as episode of infection that needed hospitalization) was evident in 9.6% of patients (239/2.491); one third of them (35%) occurred during the previous year (data available in 155/239 patients). Regression multivariate analysis showed that patients with a history of serious infection were more likely to be older (2.2% increase per year), having been exposed to bDMARDs (OR 2.7, 95% C.I.=1.9–3.8) and having comorbidities such as COPD (OR 2.6, 95% C.I.=1.5–4.4) and coronary artery disease (OR 1.9, 95% C.I.=1.1–3.3). The prevalence of HBV infection, defined as positive HBsAg, was estimated at 2.1% (**Figure 7**), while 10% of patients had evidence of past/resolved HBV infection (HBsAg- and anti-HBC+). Chronic HCV infection (anti-HCV+ and HCV RNA+) was less frequent (0.7%) while 0.5% of patients had evidence of past HCV infection (anti-HCV+ and HCV RNA−).

Regarding tuberculosis, history of active tuberculosis was reported by 1.5% of patients whereas data for LTBI diagnosis by TST or an IGRA were available from 1.565 and 300 patients, respectively. A positive TST was reported in 13% (204/1565) and a positive IGRA in 15.3% (46/300) of patients (**Figure 7**).

A history of HZ was elicited in 6.2% of patients (n=154, **Figure 7**). Vaccination against pneumococcus in the past was reported in 36% of patients while more than half (52%) had been vaccinated against influenza, but only one-third during the previous year (32%). Nine percent of patients had been vaccinated against HBV (**Figure 7**).

##### Other comorbidities

Other important comorbidities in daily clinical practice such as neoplastic diseases (current or past), depression (defined by antidepressant therapy), osteoporosis (defined by anti-osteoporotic therapy and/or history of osteoporotic fractures) and COPD were reported in 5%, 12%, 29% and 6% of patients, respectively.

## DISCUSSION

This is one of the largest studies in the literature providing real-life data regarding the current disease characteristics, treatment patterns and comorbidities of RA patients. Although there have been a number of well-designed cross-sectional studies and registry data^11–15^ that have provided useful information on these characteristics, few studies have reported detailed data on all aspects of the disease, various comorbidities and its current management.

Updated real-world data regarding RA are also limited from Greece, where the available studies have focused mainly on biologic therapies^7,9,10^ and their side effects.^8^ In the first phase of this 3-year long prospective study, a cross-sectional evaluation of baseline patient and disease characteristics, as well as treatment patterns and comorbidities from approximately 2.500 RA patients was performed.

Overall the patient characteristics from this RA cohort did not differ significantly from recent RA cohorts.^13^ The majority of patients were women (∼80%) with long disease duration (∼10 years) and a slightly higher mean patient age (∼63 years) compared to the other cohorts. Regarding their working status, only one out of four patients (28%) were still at work; a rate that is similar to what has been recently reported in the US CORRONA database (31%, mean age: 59 years).^14^ Interestingly, even in patients younger than 67 (the usual age of retirement in Greece), one-third had already retired from their work, indicating probably the chronic detrimental effect of RA in their daily function and a significant financial loss for the patients and the society.

Reflecting on the worldwide obesity pandemic, almost two-thirds (68%) of our RA patients were overweight (BMI>25 Kg/m^2^), a finding similar to other recent RA cohorts from Western countries (54–69%).^13^ Approximately, one out of five patients (19%) were active smokers at the time of evaluation, which in combination to their increased weight may contribute to the increased cardiovascular risk of these patients. One-third of patients (33%) reported alcohol use which is somewhat lower to that reported in the US CORRONA database (43%).^14^ Likewise, the proportion of patients drinking more than 2 drinks a week was lower (6%) compared to RA patients from the US database (22%: ≥1 drink/week).^14^

Interestingly, only half of our patients (52%) were positive for anti-CCP antibodies or RF, a rate lower than that reported from other recent RA cohorts (72–84%).^13^ Similarly low rates of seropositivity were reported from another Greek RA cohort recently,^10^ probably indicating a different serological phenotype of RA patients in Greece. Nevertheless, more studies are needed in order to confirm these findings.

A cross-sectional real-life study provides the unique opportunity to have an accurate picture of the current disease activity in the era of modern therapeutics. The mean disease activity, as measured by the DAS28-ESR score, was 3.4 reflecting a moderate to low disease activity of this cohort. These results are in agreement with recent values reported in the international COMORA study (n=3.920)^15^ and the US CORRONA (n=24.989)^14^ database (mean DAS28-ESR: 3.7 and 3.4, respectively). Despite these encouraging results, almost half of our patients (48%) had still moderate or high disease activity, which according to the most recent treatment guidelines^16^ or recommendations^17,18^ necessitate a change in therapy. Similar or even higher rates of residual disease activity have been recently reported from different RA cohorts worldwide (58–76%),^13^ emphasizing the need for more aggressive therapeutic strategies in order to achieve the predefined goals according to the universally accepted treat to target concept.^18^

On the other hand, the functional assessment of our patient population by the HAQ score showed a relatively low mean value (0.48) close to what has been reported in the US CORRONA registry (0.4)^14^ but lower to that from other cohorts.^13,15^ It is unclear at the moment if this finding is due to a less destructive course of RA in Greece (collaborated by the lower seropositivity rate, as discussed above) and/or it is reflecting the advantageous effect of modern therapies.

The large number of patients included in this RA cohort (n=2.491), provided also the opportunity to study the current treatment trends. At the time of evaluation, 82% of the patients were being treated with csDMARDs and, as expected, MTX was the most commonly used one (77% among csDMARD treated patients). Although combination csDMARD therapy is recommended after failure of csDMARD monotherapy,^16,17^ few data exist for their actual use in daily clinical practice. In this cohort, only 11% of RA patients were treated with csDMARD combinations. The most commonly used combination was that of MTX and HCQ while the triple csDMARD combination of MTX, HCQ and SSZ was rarely utilized.

Regarding bDMARDs, 42% of the participants were receiving a biologic agent at the time of recruitment. Obviously, this finding does not reflect the proportion of bDMARD-treated patients in the total population of RA patients in Greece, given that, based on data derived from the national administrative electronic prescription database, this has been recently estimated to be approximately 15%.^19^ This over-presentation of bDMARD-treated patients is a common observation in several registries and is probably attributed to the fact that the majority of the patients are recruited by tertiary and academic centers as is the case in our study (96%).

This over-presentation may also explain the high proportion of patients treated with non-TNFi (i.e., rituximab, tocilizumab, abatacept), given that most of these therapies were administered intravenously during the study period (2015–16). Forty-seven percent (47%) of the bDMARD-treated patients were receiving non-TNFi at the time of evaluation, when the respective proportion in CORRONA registry was only 13%.^14^ In support of this assumption, in the Greek prescription electronic database, the proportion of non-TNFi therapies was 27%.^19^ On the other hand, it should be mentioned that most of the published studies were actually initiated well before ours, when the only available bDMARDs were those of TNFi class plus rituximab.

bDMARDs as monotherapy was used overall in approximately 11% of patients which represents one out of four patients treated with bDMARDs (26%). This rate of bDMARD monotherapy was higher than that reported by the US CORRONA database (19%)^20^ and close to that of the recent Swiss registry (27%).^21^

It is well recognized nowadays that a substantial proportion of morbidity and mortality in RA patients is attributed to comorbidities, mainly cardiovascular and respiratory diseases.^13,22,23^ In alignment with these findings, a high prevalence of several comorbidities was found in our cohort. Regarding the prevalence of cardiovascular disease risk factors, a high prevalence of arterial hypertension (42%), hyperlipidemia (33%), obesity (BMI > 30 kg/m^2^, 26%), diabetes (15%) and active smoking (19%) was found, with 40% of patients having at least two of the above risk factors. Similarly high rates of hypertension in Greek RA patients has been reported in the past^24^ and recently in another Greek RA cohort (44%) compared to a matched age- and gender-population control group (34%).^25^

In a recent analysis, based on the electronic prescription database by Sfikakis et al, of all RA patients treated with bDMARDs in Greece (n=9.824, females: 79%, mean age: 61 years),^19^ the prevalence of the main cardiovascular disease risk factors such as hypertension, hyperlipidemia and diabetes was quite similar to that reported in our study for bDMARD treated (n=1.036) patients (41% vs. 42%, 35% vs. 26% and 13% vs. 12%, respectively). These findings underscore the validity of our findings from our patient cohort and emphasize the need for their early identification and management in order to prevent cardiovascular events.

In our study, as described and in other registries, infections were found to be a major comorbidity. Approximately one out of ten patients had a history of serious infection, with one third of the events occurred during the last year before registration. The prevalence of chronic hepatitis B and C was 2.1 and 0.7%, respectively. These rates are similar to those reported in other epidemiological studies from Greece^26^ and close to what has been reported in the international COMORA study (HBV: 3%, HCV: 2%).^15^

Although Greece is generally considered a low TB incidence country (incidence rate: 4.5–30/100.000 patients),^27,28^ approximately 13–15% of patients had evidence of LTBI (by TST or IGRA). In a recent study, Houben et al estimated the prevalence of LTBI at 16.3% for the general population in Eastern Mediterranean region and 13.7% in Europe.^29^ Taken all these together and given the proven efficacy of strategies in reducing the risk for TB reactivation in rheumatic patients,^30^ screening for LTBI prior to bDMARD initiation should continue to be an integral part of our daily clinical practice.

Vaccination remains one of the most efficacious and low-cost strategies for the prevention of various bacterial and viral infections in rheumatic patients treated with immunosuppressives.^31^ In agreement with the findings from the international COMORA study,^15^ only one third of RA patients had been vaccinated against pneumococcus (36%) or influenza virus (32% the previous year), emphasizing the urgent need for better education of health care providers and patients towards the goal of universal vaccine coverage.

Major strengths of our study included its large number of patients (n=2.491), its nationwide multicenter design, with hospital-based outpatient clinics, as well as private offices participating, and a geographic coverage that includes most of the regions of the country and almost all the main referral centers. Moreover and unlike other registries, this study included a large number of patients treated with non-TNFi bDMARDs as well as a large number of patients treated with csDMARDs (either as monotherapy or combination therapy). Additionally, our study provided detailed information regarding the profile of various comorbidities of RA patients in Greece today. Given the impact of these conditions on the morbidity and mortality of patients with RA, we consider that these data will contribute in the better design of screening and preventive strategies for this vulnerable population.

Our study has also certain limitations. The most important is that the majority of recruited patients were from referral centers (“referral bias”), which may have caused an over-representation of more severe cases. Nevertheless, the comparison of the patients’ main characteristics with the national administrative electronic database did not reveal other significant differences. Obviously as in other registries and RA cohorts, there was no universal protocol for treatment or screening for the major comorbidities and this could add to the heterogeneity of our results. It should be noted though that since 2009, the Greek Rheumatology Society is regularly issuing updated recommendations for the management of RA and its therapeutic algorithm has been implemented in the obligatory electronic prescription of anti-rheumatic therapies during the last 5 years. Finally, missing data is always a limitation in observational studies; nevertheless in our study completeness of data was close to 90% and thus we consider that this was not an issue in the present analysis.

In conclusion, the results of this large cross-sectional study provide novel data regarding the current profile of RA in Greece as well as changing trends in its management. In addition, our study highlights the high prevalence of certain comorbidities and the unmet need for wider dissemination of preventive strategies such as vaccination in this population.

## References

[1] Smolen JS Aletaha D McInnes IB. Rheumatoid arthritis. Lancet 2016;388:2023–38. 27156434 10.1016/S0140-6736(16)30173-8

[2] Alamanos Y Voulgari PV Drosos AA. Incidence and prevalence of rheumatoid arthritis, based on the 1987 American College of Rheumatology criteria: a systematic review. Semin Arthritis Rheum 2006;36:182–8. 17045630 10.1016/j.semarthrit.2006.08.006

[3] Andrianakos A Trontzas P Christoyannis F Kaskani E Nikolia Z Tavaniotou E Prevalence and management of rheumatoid arthritis in the general population of Greece--the ESORDIG study. Rheumatology (Oxford) 2006;45:1549–54. 16690763 10.1093/rheumatology/kel140

[4] Drosos AA Alamanos I Voulgari PV Psychos DN Katsaraki A Papadopoulos I Epidemiology of adult rheumatoid arthritis in northwest Greece 1987–1995. J Rheumatol 1997;24:2129–33. 9375871

[5] Anagnostopoulos I Zinzaras E Alexiou I Papathanasiou AA Davas E Koutroumpas A The prevalence of rheumatic diseases in central Greece: a population survey. BMC Musculoskelet Disord 2010;11:98. 20504294 10.1186/1471-2474-11-98 PMC2890601

[6] Guillemin F Saraux A Guggenbuhl P Roux CH Fardellone P Le BE Prevalence of rheumatoid arthritis in France: 2001. Ann Rheum Dis 2005;64:1427–30. 15800010 10.1136/ard.2004.029199 PMC1755224

[7] Flouri I Markatseli TE Voulgari PV Boki KA Papadopoulos I Settas L Comparative effectiveness and survival of infliximab, adalimumab, and etanercept for rheumatoid arthritis patients in the Hellenic Registry of Biologics: Low rates of remission and 5-year drug survival. Semin Arthritis Rheum 2014;43:447–57. 24012040 10.1016/j.semarthrit.2013.07.011

[8] Lampropoulos CE Orfanos P Bournia VK Karatsourakis T Mavragani C Pikazis D Adverse events and infections in patients with rheumatoid arthritis treated with conventional drugs or biologic agents: a real world study. Clin Exp Rheumatol 2015;33:216–24. 25664400

[9] Vassilopoulos D Delicha EM Settas L Andrianakos A Aslanidis S Boura P Safety profile of repeated rituximab cycles in unselected rheumatoid arthritis patients: a long-term, prospective real-life study. Clin Exp Rheumatol 2016;34:893–900. 27383049

[10] Thomas K Flouri I Repa A Fragiadaki K Sfikakis PP Koutsianas C High 3-year golimumab survival in patients with rheumatoid arthritis, ankylosing spondylitis and psoriatic arthritis: real world data from 328 patients. Clin Exp Rheumatol 2017. 29148406

[11] Souto A Maneiro JR Gomez-Reino JJ. Rate of discontinuation and drug survival of biologic therapies in rheumatoid arthritis: a systematic review and meta-analysis of drug registries and health care databases. Rheumatology (Oxford) 2016;55:523–34. 26490106 10.1093/rheumatology/kev374

[12] Hudson M Tascilar K Suissa S. Comparative effectiveness research with administrative health data in rheumatoid arthritis. Nat Rev Rheumatol 2016;12:358–66. 27080692 10.1038/nrrheum.2016.34

[13] Michaud K Berglind N Franzen S Frisell T Garwood C Greenberg JD Can rheumatoid arthritis (RA) registries provide contextual safety data for modern RA clinical trials? The case for mortality and cardiovascular disease. Ann Rheum Dis 2016;75:1797–805. 26857699 10.1136/annrheumdis-2015-208698

[14] Solomon DH Reed GW Kremer JM Curtis JR Farkouh ME Harrold LR Disease activity in rheumatoid arthritis and the risk of cardiovascular events. Arthritis Rheumatol 2015;67:1449–55. 25776112 10.1002/art.39098 PMC4446181

[15] Dougados M Soubrier M Antunez A Balint P Balsa A Buch MH Prevalence of comorbidities in rheumatoid arthritis and evaluation of their monitoring: results of an international, cross-sectional study (COMORA). Ann Rheum Dis 2014;73:62–8. 24095940 10.1136/annrheumdis-2013-204223 PMC3888623

[16] Singh JA Saag KG Bridges SL Jr. Akl EA Bannuru RR Sullivan MC 2015 American College of Rheumatology Guideline for the Treatment of Rheumatoid Arthritis. Arthritis Rheumatol 2016;68:1–26. 26545940 10.1002/art.39480

[17] Smolen JS Landewe R Bijlsma J Burmester G Chatzidionysiou K Dougados M EULAR recommendations for the management of rheumatoid arthritis with synthetic and biological disease-modifying antirheumatic drugs: 2016 update. Ann Rheum Dis 2017;76:960–77. 28264816 10.1136/annrheumdis-2016-210715

[18] Smolen JS Breedveld FC Burmester GR Bykerk V Dougados M Emery P Treating rheumatoid arthritis to target: 2014 update of the recommendations of an international task force. Ann Rheum Dis 2016;75:3–15. 25969430 10.1136/annrheumdis-2015-207524 PMC4717393

[19] Sfikakis PP Bournia VK Sidiropoulos P Boumpas DT Drosos AA Kitas GD Biologic treatment for rheumatic disease: real-world big data analysis from the Greek country-wide prescription database. Clin Exp Rheumatol 2017;35:579–85. 28281458

[20] Pappas DA Reed GW Saunders K John A Shewade A Greenberg JD Characteristics Associated with Biologic Monotherapy Use in Biologic-Naive Patients with Rheumatoid Arthritis in a US Registry Population. Rheumatol Ther 2015;2:85–96. 27747494 10.1007/s40744-015-0008-9 PMC4883255

[21] Gabay C Riek M Scherer A Finckh A. Effectiveness of biologic DMARDs in monotherapy versus in combination with synthetic DMARDs in rheumatoid arthritis: data from the Swiss Clinical Quality Management Registry. Rheumatology (Oxford) 2015;54:1664–72. 25922549 10.1093/rheumatology/kev019

[22] Young A Koduri G Batley M Kulinskaya E Gough A Norton S Mortality in rheumatoid arthritis. Increased in the early course of disease, in ischaemic heart disease and in pulmonary fibrosis. Rheumatology (Oxford) 2007;46:350–7. 16908509 10.1093/rheumatology/kel253

[23] Pinheiro FA Souza DC Sato EI. A Study of Multiple Causes of Death in Rheumatoid Arthritis. J Rheumatol 2015;42:2221–8. 26472415 10.3899/jrheum.150166

[24] Karvounaris SA Sidiropoulos PI Papadakis JA Spanakis EK Bertsias GK Kritikos HD Metabolic syndrome is common among middle-to-older aged Mediterranean patients with rheumatoid arthritis and correlates with disease activity: a retrospective, cross-sectional, controlled, study. Ann Rheum Dis 2007;66:28–33. 16793841 10.1136/ard.2006.053488 PMC1798406

[25] Protogerou AD Panagiotakos DB Zampeli E Argyris AA Arida K Konstantonis GD Arterial hypertension assessed “out-of-office” in a contemporary cohort of rheumatoid arthritis patients free of cardiovascular disease is characterized by high prevalence, low awareness, poor control and increased vascular damage-associated “white coat” phenomenon. Arthritis Res Ther 2013;15:R142. 24286134 10.1186/ar4324 PMC3978881

[26] Gogos CA Fouka KP Nikiforidis G Avgeridis K Sakellaropoulos G Bassaris H Prevalence of hepatitis B and C virus infection in the general population and selected groups in South-Western Greece. Eur J Epidemiol 2003;18:551–7. 12908721 10.1023/a:1024698715741

[27] European Centre for Disease Prevention and Control/WHO Regional Office for Europe Tuberculosis surveillance and monitoring in Europe 2017. Accessed at: ecdc.europa.eu/sites/portal/files/documents/ecdc-tuberculosis-surveillance-monitoring-Europe-2017-WEB.pdf on January 5, 2018.

[28] Lytras T Spala G Bonovas S Panagiotopoulos T. Evaluation of tuberculosis underreporting in Greece through comparison with anti-tuberculosis drug consumption. PLoS One 2012;7:e50033. 23185524 10.1371/journal.pone.0050033 PMC3503712

[29] Houben RM Dodd PJ. The Global Burden of Latent Tuberculosis Infection: A Re-estimation Using Mathematical Modelling. PLoS Med 2016;13:e1002152. 27780211 10.1371/journal.pmed.1002152 PMC5079585

[30] Carmona L Gomez-Reino JJ Rodriguez-Valverde V Montero D Pascual-Gomez E Mola EM Effectiveness of recommendations to prevent reactivation of latent tuberculosis infection in patients treated with tumor necrosis factor antagonists. Arthritis Rheum 2005;52:1766–72. 15934089 10.1002/art.21043

[31] Thomas K Vassilopoulos D. Immunization in patients with inflammatory rheumatic diseases. Best Pract Res Clin Rheumatol 2016;30:946–63. 27964798 10.1016/j.berh.2016.10.009

